# Spirituality and spiritual care in in the context of nursing education in South Africa

**DOI:** 10.4102/curationis.v38i1.1471

**Published:** 2015-12-17

**Authors:** Sandhya Chandramohan, Raisuyah Bhagwan

**Affiliations:** 1 Greys Nursing Campus, KwaZulu-Natal College of Nursing, South Africa; 2 Community Health Studies, Child and Youth Care Programme, Durban University of Technology, South Africa

## Abstract

**Background:**

In order for nursing education to prepare nurses for holistic patient care, it is critical that educators become more aware of the religious and spiritual dimensions in patient care and be able to provide adequate knowledge and skills for nurses to offer spiritually-based care in an ethical way. Furthermore, spiritual care is an essential component in the nursing context, as nurses have to care for patients who may often turn to the spiritual dimension to cope and heal. These aspects are important issues to be considered in planning what should be taught as part of spiritual care.

**Objectives:**

This paper presents findings from a study on nursing practitioners’ views on the role of spiritual care in nursing practice and whether current nursing education has integrated this dimension into teaching.

**Method:**

A descriptive survey using a cross-sectional design with 385 nurses was conducted between December 2012 and February 2013. Participants were recruited through multistage random sampling. Data analysis was undertaken using SSPS 0.20.

**Results:**

All the participants (*n* = 385) concurred that spiritual care was a salient component of holistic patient care. They however stated that the primary barriers to providing spiritual care related to uncertainty on how to provide this type of care, and a lack of educational preparedness for this role.

**Conclusion:**

The study found that nurses were very accepting of the need for spiritual care as part of their nursing role but that nursing education had not paid adequate attention to integrating this dimension into the nursing curriculum.

## Introduction

### Background

Research on health, well-being and spirituality has grown in the Western context, with studies showing that spirituality is an important part of a patient’s life when confronted with illness (Koenig 2009:283). Empirical work, in particular, has documented that spirituality becomes particularly salient when patients have to deal with HIV and AIDS, cancer and heart disease (Koenig 2009:283). Despite the growing body of literature abroad (Barlow 2011; Graham 2008; McSherry & Jamieson 2010; Nixon & Narayanasamy 2010), research and literature on spirituality and spiritual care is sparse in South Africa.

It has been argued that a failure to incorporate spirituality into nursing care by not addressing the spiritual needs of patients is unethical as spirituality is a part of being human (Miner-Williams 2006:811) and contradicts holistic patient care. The lack of formal educational preparedness on spirituality and spiritual care appears to be the primary factor that has rendered nurses unprepared to deliver spiritual care (Barlow 2011:1).

Several authors have noted that nursing education has provided few opportunities for the inclusion of spirituality and spiritual care (Dunn 2008:4; Molzahn & Shields 2008:25; O’ Shea et al. 2011:36). This void leaves the nurse unprepared to meet the challenges of providing therapeutic spiritual care for patients and their families. Both Lubbe (2008:66) and Dunn (2008:4) assert that there is a need for spirituality and spiritual care activities to be included in the nursing curricula.

### Problem statement

There exists a huge empirical gap in South Africa on spirituality and spiritual care in nursing practice and nursing education. Internationally, however, studies have burgeoned focusing on the views of nursing practitioners and academics with regard to spirituality and spiritual care in nursing practice and education.

## Aims of the study

This study sought to explore the views of nursing practitioners with regard to the role of spirituality in nursing practice and education. It also sought to explore whether nursing education included spiritual care as part of the academic curriculum for nursing students. This article presents findings on the latter.

## Objectives

The objectives of the study were:

- To explore the views of nurses at public hospitals in KwaZulu-Natal regarding the role of spirituality and spiritual care in nursing practice.
- To investigate whether nurses utilise spiritually-based activities in nursing.
- To investigate whether current education and training has prepared nurses for spiritual care practice.

## Definitions of key concepts

**Spirituality:** The presence of a relationship with a Higher Power, a response to a deep and mysterious human yearning for self-transcendence and surrender, a yearning to find our place and the search for the existential (McSherry & Jamieson 2010:1757; O’ Brien 2011:4).

**Religion:** The membership of and adherence to the practice of a particular faith, tradition or sect (Barlow 2011:1).

**Holistic care:** Care of the mind, body and soul/spirit (Lundberg & Kerdonfag 2010:1121).

**Professional nurse:** A nurse who is educated and competent to practice comprehensive nursing, assumes responsibility for independent decision-making, and is licenced as a professional nurse under the *South African Nursing Act* (SANC 2013).

## Contribution to the field

To meet the spiritual needs of patients, a nurse must be knowledgeable about issues surrounding their religion and spirituality, especially issues that interface with their health, illness and recovery. Incorporating spiritual care into nursing education will effectively prepare nurses to deal with the complexity of providing personalised spiritual care in an increasingly diverse society. Moreover, it will ensure the provision of holistic care that integrates spirituality together with the physical and psychological dimensions of patient care.

Furthermore, spirituality will also be considered as an important pillar alongside the physical and psychological dimensions in nursing care. Spiritual care embraces respect, offering comfort, listening to the patient, instilling hope, prayer and holding the patient’s hand (McSherry & Jamieson 2010:1757). In nursing it is important to empower nurses with adequate knowledge and skills that will enable them to offer spiritual care and foster hope, purpose and meaning in the lives of those who are ill.

## Conceptual framework

Confronted with helplessness and anxiety experienced as a result of illness many patients seek meaning, hope, love and comfort through human relationships or a transcendent dimension with God (O’ Brien 2011:2). Nixon, Narayanasamy and Penny (2013:7) state that spirituality comes into focus when an individual faces emotional stress, physical illness or death. Spiritually-based activities such as prayer and meditation decrease the fear of death, increase comfort and support a positive perspective of death amongst gravely ill patients (Caramanzana & Wilches 2012:295; Laukhuf & Werner 1998 in Nixon & Narayanasamy 2010:2260).

The present study adopted the Human-To-Human Relationship Model of Travelbee (Hutchinson 1998) as a guide for quality spiritual patient care and education. According to Travelbee (Hutchinson 1998), nurses do not only seek to alleviate physical pain or render physical care, they minister to the whole person. The Human-To–Human Relationship Model rests on the notion that nursing is fulfilled through a human-to-human relationship, which also considers the spiritual beliefs and needs of the patient (O’ Brien 2011:2). According to Frankl (2006:121), ‘the primary motivation of humankind is his search for meaning in life’; further stating that this search for meaning helps a person to cope with suffering and the stressful events of daily living. Spirituality and religion forms a primary means by which patients can find meaning through their illness and suffering; nurses need to be knowledgeable about how to bring this element into nursing care.

## Literature review

Spirituality refers to the presence of a relationship with a Higher Power, a response to a deep and mysterious human yearning for self-transcendence and surrender, a yearning to find our place and the search for existential (McSherry & Jamieson 2010:1757; O’ Brien 2011:4). Although scholars have argued that religion and spirituality are inseparable and that both constructs can be used interchangeably (Penman 2012:135; Rieg, Mason & Preston 2006:249), others view spirituality as a broader concept that transcends religion (Lubbe 2008:8; McSherry & Jamieson 2010:1757; Nixon & Narayanasamy 2010:2260; O’ Brien 2011:4). These scholars have therefore advocated that spiritual care be included in nursing education.

Research has shown that nurses who are hesitant to provide spiritual care are those who are not in touch with their own spirituality, are confused about the role of the nurse in providing spiritual care, have a lack of knowledge regarding what spiritual care is, and fear imposing their own spiritual or religious preference on patients (Callister et al. 2004:106). The South African Nursing Council’s (SANC 2012) philosophy of nursing practice and education does not provide a specific definition of holistic nursing care, which has led to different interpretations in its application at patient care level.

The basic premise underlying the Scope of Practice of the Professional Nurse (R2598 of November 1998, as amended) is that nurses are concerned with the human being as a holistic being (Tjale & De Villiers 2008:10). According to the Scope of Practice, the expected outcome of the nurse-patient encounter is that the professional nurse will render comprehensive holistic nursing care, as stated in the White Paper (Tjale & De Villiers 2008:11).

Despite a growing body of evidence that indicates the importance of spiritual care in nursing practice, there is little research related to whether spirituality is considered in nursing education (Chism & Magnan 2009:603). A survey was conducted with 223 student nurses in Michigan to investigate their personal level of spirituality and the training received on spiritual care. It was found that 96 (43%) of the student nurses considered themselves spiritual and 190 (85%) acknowledged having a religious affiliation. Only 23 (13%) however indicated being satisfied with their training on spiritual care (Chism & Magnan 2009:600). These findings reflect a void in professional preparedness abroad despite the growing literature that supports the importance of spirituality in nursing care (Chism & Magnan 2009:600; Graham 2008:33; Penman 2012:135). Hanson and Andrew (2012:354) argue that it is ethically wrong to neglect the spiritual needs of patients, stating that such neglect should carry the same consequences as neglect of physical pain.

Graham (2008:33) examined the perceptions of European nursing students (*n* = 24) on how prepared they were in terms of assessing patients’ spiritual needs. Quantitative data was collected after they participated in a four-hour spirituality seminar. The seminar was rated as being positive and common spiritual care interventions were identified by about 80% of the students (*n* = 24) after the seminar. The interventions seen as important for education included praying with the patient, providing support in the clinical setting, practicing physical presence, and assuring patients of God’s forgiveness (Graham 2008:40).

Nathan, Wylie and Marsella (2001) stated that there are fundamental difficulties that nurses experience when trying to meet a patients’ spiritual needs. This is rooted in the ignorance of what spirituality is and how to deal with spiritual issues when they arise. Nathan et al. (2001) opined that whilst nurses were good at dealing with ritualistic aspects such as dietary needs, they were unprepared to meet spiritual concerns such as ‘Why is this happening to me?’ and ‘How will I cope?’; these authors therefore argued that there was a need for knowledge on how to render spiritual care.

Amoah (2011:355) refers to the importance of valuing spiritual experience in nursing care. The author (2011) points out that religion and spirituality is interlinked with the code of dressing; dietary issues; bodily touch; views about treatment; prayers, rituals, sacraments, symbols and holy books; rituals after death and emotional expressions, all of which should be considered in nursing education. Spiritual care competence thus ensures that nurses serve as companions on a journey which engenders hope and facilitates healing in the face of illness and hopelessness.

About 90% of the sample in the current study indicated that the following deserved attention in education, namely, knowledge on whether and how to pray with a patient; appropriate use of therapeutic touch such as holding the patient’s hand; active listening skills; when and how to refer a patient to a priest or religious leader; and how to convey acceptance of the patient’s spiritual beliefs.

## Research design and method

### Design

A quantitative descriptive design was used to survey professional nurses (*n* = 385) at selected public hospitals through a process of multiphase random sampling.

### Materials

A total of 550 questionnaires were distributed and 385 were returned. The questionnaire included two scales. The first one was The Role of Religion and Spirituality in Social Work Practice, developed by Prof. Sheridan, which was adapted for nurses in this study; whilst the second scale was The Spirituality and Spiritual Care Rating Scale (SSCRS), utilised by McSherry and Jamieson (2010:1757). The second scale examined spirituality and spiritual care in nursing practice and nursing education. The researchers granted permission for the use of these scales for the purposes of this study.

These scales, together with the researcher’s own questions gave birth to a new questionnaire. This comprised of closed-ended, open-ended and Likert type matrix questions. The following themes reflected the sub-sections of the questionnaire: demographic details; views with regard to the role of spirituality in nursing practice; the salience of spirituality to patients; spiritual-based interventions; and spirituality in education.

### Data collection

Survey questionnaires were delivered to each of the hospitals by the researcher. Whilst some questionnaires were collected at the end of the same day, some required follow-up visits to collect questionnaires that were completed later. All questionnaires were coded from 1 to 385. Data was entered onto a coded spread sheet using the SPSS version 20.0.

### Data analysis

The data was analysed using the statistical software SPSS version 20.0. Descriptive statistics and inferential statistics were applied to the data.

Descriptive statistics describe the organisation and summary of quantitative data and then determine whether the scores on different variables are related to each other (Lind, Marchal & Mason 2004:6). Cronbach’s alpha scoring, for all sections of the questionnaire, was more than 0.70. An alpha score of more than 0.70 indicates a high level of reliability (Brink, Van der Walt & Van Rensburg 2012:172). Factor analysis identified underlying variables that explained the pattern of correlations within a set of observed variables.

Pearson’s correlation tests were also conducted. Pearson’s *r*-value indicates the strength of the relationship between two or more variables (Brink et al. 2012:172). Pearson’s correlation was used to analyse the relationships between the following variables: gender, age, educational level, spiritual beliefs, and years of experience. Chi-square testing was also undertaken.

### Context of the study

The population for this study included all 25 440 professional nurses in KwaZulu-Natal who were on the register of the South African Nursing Council (SANC 2012). As it was impossible to survey all the nurses due to time and financial constraints, an appropriate sampling strategy (multistage random sampling) was used, after consultation with a registered statistician. This involved the successive random sampling of units, beginning with the largest group and progressing to smaller units (Burns & Grove 2008:351; Polit & Beck 2008:347).

Multistage random sampling was operationalised in the following way: In stage one KwaZulu-Natal was divided into its 11 districts: eThekwini, uMgungundlovu, UGu, uThukela, uMzinyathi, Amajuba, Zululand, uThungulu, iLembe, Sisonke and uMkhanyakude. Seven of the 11 districts were selected as they have public hospitals that offer practical training for the nursing diploma programme via eThekwini, uMgungundlovu, UGu, uMzinyathi, Amajuba Zululand, and uThungulu.

In stage two, five of these seven districts identified in stage one were selected as these districts have regional/tertiary level public hospitals: eThekwini, uMgungundlovu, uThungulu, UGu and Amajuba. Only one hospital per district was utilised. In the final stage, a sample of 37% of professional nurses per hospital was surveyed as per consultation with the statistician. A total of 550 questionnaires were distributed and 385 questionnaires were returned, which yielded a 77% return rate, which was beyond the norm for a good response rate of between 50% and 60% (Polit & Beck 2008:187).

## Results

The survey found that 296 participants (77.1%) received some training on spiritual care in their student years, whilst 88 (22.9%) had received no training at all. Participants were then asked to assess their level of satisfaction with the information received on spirituality. A total of 212 participants (64.2%) reported being somewhat satisfied with the training that they had received on spirituality and spiritual care. However, 118 participants (35.8%) indicated being dissatisfied with the information they received on spirituality during their training.

Nurses were then asked about their training on spirituality and spiritual care after they had qualified. A total of 304 participants (80.2%) indicated that they had no post-qualification training related to spiritual care, whilst 75 (19.8%) indicated that they had received some training or attended workshops that focused on spiritual care.

### Content to be included in training courses on spirituality and spiritual care

Participants were asked to comment on what they would like to be included in training. Responses suggested a need for more spirituality workshops and that subject content should include spiritual care content, prescribed and recommended textbooks on spirituality, and seminars on spirituality and spiritual care. As can be seen from Table 1, the sample agreed that patients benefit from spiritual care when faced with illness, psychological distress and emotional difficulties. In addition, 90% of the sample indicated that the following deserved attention in education, namely, knowledge on whether and how to pray with a patient; appropriate use of therapeutic touch such as holding the patient’s hand; active listening skills; when and how to refer patient to a priest or religious leader; and how to convey acceptance of the patient’s spiritual beliefs.

**TABLE 1:** Salience of spirituality to patients.

Statement	Agree	Uncertain	Disagree
All patients have their own belief in spirituality	93.5	3.1	3.4
As individuals grow, life experiences increase their spiritual maturity	82.3	13.0	4.7
Spiritual participation helps protect patients against depression	82.1	13.8	4.2
Patients who are abused or neglected may especially benefit from spiritual beliefs or practices	81.3	13.0	5.7
Religious beliefs provide guidelines for behaviours that are beneficial to patients	80.5	14.8	4.7
Some patients are exceptionally spiritually mature or gifted	78.7	15.8	5.5
Terminally ill patients search for meaning and purpose in life	69.4	18.7	11.9
It is not unusual for some patients to have spiritual experiences that influence their lives	67.6	19.3	13.1
Some patients do not have the cognitive abilities to reflect on spiritual matters	65.8	22.2	12.0
Some patients experience problems or anxiety due to spirituality that goes unnoticed by nurses	57.9	26.5	15.6
Hospitalisation is a time of spiritual awareness	52.3	24.5	23.2
As patients grow older, they lose their natural connection to spirituality Appendix 1	20.8	14.3	64.8

The correlation value between ‘nursing education should include content related to spiritual diversity’ and ‘nurses should have more knowledge about spiritual care in nursing’ was 0.253. This suggests strong support for the inclusion of spirituality and spiritual care in nursing practice and the need for more knowledge related to issues of spiritual diversity. Spiritual diversity is interrelated, not only with religious diversity and an understanding of the different faith traditions, but also human diversity issues such as gender and sexuality. This was further supported in the correlation found between ‘nursing education should include content related to spiritual diversity’ and ‘It is important for nurses to have knowledge about different religious faiths and traditions’ (a score of 245), which emphasises that nurses need more information, as part of their education, on spiritual diversity and how issues of health and recovery differ across diverse spiritual perspectives. Attention to alternative and indigenous therapies embedded in diverse cultures should also form part of nurses’ education.

A positive correlation was also found between ‘nursing education should include content on how to deal with spiritual issues in nursing’ and ‘I believe nurses can provide spiritual care by showing kindness, genuine concern and cheerfulness when dealing with patients’ (a score of 442). This reflects that kindness and showing genuine concern are important threads of spiritually-based nursing care and should be emphasised as the core values underpinning spiritual care.

### Potential topics on spirituality in nursing education

The survey also explored the views of nurses with regards to what topics should be included in their education. They indicated that the following topics should be included in their education, namely, information on different religious faiths and traditions; spiritual diversity; the positive/beneficial role of spiritual beliefs and practices in the lives of patients; salience of spirituality to patients; medical personnel responsible for providing spiritual care; spiritual beliefs related to health and illness; physical and social environments that promote spiritual well-being; the role of spiritual healers in spiritual nursing care; and spiritual interventions.

Spiritual care interventions that needed to be integrated into education included how to pray with a patient; providing quiet time and privacy; facilitating and validating of a patient’s feelings; instilling hope and offering comfort; being physically present; showing respect; find meaning in illness; and finding purpose in one’s life. Effective and comprehensive educational programmes lay the foundation for meaningful learning (Lind, Sendelbach & Steen 2011:89). Yong et al. (2011:280) stated that training courses have been effective in preparing nurses to deliver spiritual-based nursing care. This is particularly important where the initial academic training has made no provision for its inclusion. It is also important that educators consider how to align current teaching content with information on spirituality and spiritual care, for example fundamental nursing care.

Scholars such as Ledger and Bowler (2013:22) highlighted what to teach by developing a spirituality training course for psychiatric nurses. Their course content included acceptance of mental illness without prejudice; maintaining respect and the dignity of the psychiatric patient; offering support and hope; and allowing psychiatric patients to verbalise their fears. The study found that spirituality is a key aspect of patient-centred holistic care, particularly in mental health care. Furthermore, the authors (2013) said that although many psychiatric patients express the importance of spirituality in their recovery, nurses often neglected this area. Psychiatric nurses acknowledged their role in meeting the spiritual needs of patients but felt that they lack confidence in this area.

## Ethical considerations

The Institution Research Ethics Committee (IREC) of the Durban University of Technology provided full ethical clearance for the study. Permission was sought from the hospital managers of the hospitals targeted and from the KwaZulu-Natal Provincial Health Research and Knowledge Management Committee. Participants completed an information and consent form which outlined details of the study and confirmed that there was no risk in terms of participating. They were reassured that they were also free to withdraw from the study, with no repercussions. Anonymity was protected as no identifying details were required. The completed questionnaires and consent forms were placed in sealed boxes by the participants. The sealed boxes were later collected by the researcher. The completed questionnaires were locked in a steel locker.

## Trustworthiness

### Reliability and validity

The Role of Religion and Spirituality in Social Work Practice Scale that was readapted for nurses was tested in three prior studies using Cronbach’s alpha. The scale demonstrated high internal consistency across all these studies, with alpha = 0.88 (Bhagwan 2002:50).

The Spirituality and Spiritual Care Rating Scale (SSCRS), which was the second scale used, also demonstrated consistent levels of reliability and validity with an original Cronbach’s alpha coefficient of 0.64 in other studies (McSherry & Jamieson 2010:1757). The SSCRS has been used in over 42 different studies in 11 countries (Lovanio & Wallace 2007:43). In addition, a Persian study using the SSCRS to assess its reliability and validity, found the instrument to be valid and reliable (Khoshknab et al. 2010:2939).

To assess the face validity of the newly developed questionnaire, it was first piloted with professional nurses to identify possible problems. The questionnaire was found to be clear and unambiguous.

## Discussion

### Outline of the results

**FIGURE 1:**
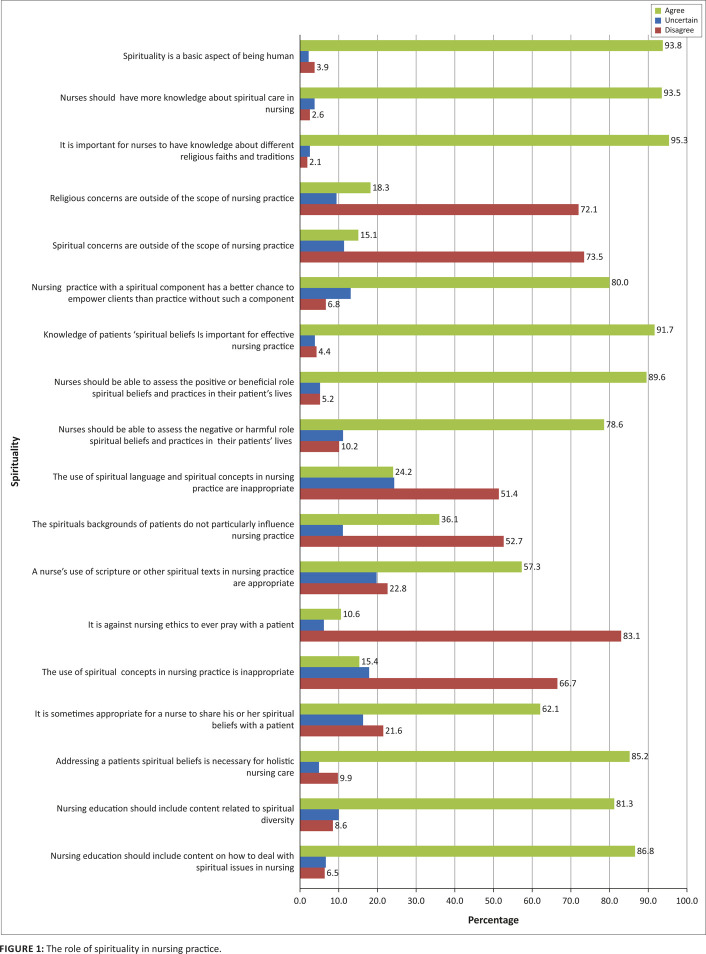
The role of spirituality in nursing practice.

In general the study found high levels of personal religiosity and spirituality amongst professional nurses; a trend evident within the South African population. Statistics South Africa (2011) reported that the majority of the South African population followed a particular faith tradition. The high level of spirituality amongst the sample in the study spilled over into their nursing role, with a majority agreeing that spirituality, spiritual care and interventions were a crucial part of holistic nursing. A relatively high response rate was found on the RRSP scale which confirmed strong support for the role of spirituality in nursing care. Whilst this may be attributed to the sample’s high level of personal spirituality, the fact that they encountered patients who brought the spiritual dimension into the nursing context may also have contributed to the high mean ratings} on the RRSP scale (Figure 1). Although about 77.1% of the sample acknowledged receiving some training on spiritual care in their student years, only 64.2% reported being somewhat satisfied with the training they received. This could be attributed to the fact that the current R425 nurse training programme has limited content on spirituality. The latter is taught in relation to culture, religion, and death and dying. The level of dissatisfaction emphasises the importance of its reconsideration in education. A small number, namely 75 (19.8%) of the participants, indicated receiving post-qualification training on spirituality and spiritual care which they found extremely beneficial and which they agreed assisted them to deliver ethically-based spiritual care. This is in direct contrast to findings from international studies where spirituality has received a more salient space in education and where conferences and seminars on spirituality have sprouted.

More than 80% of the sample (Table 1) agreed that patients benefit from spiritual care when faced with illness, psychological distress and difficulties. Prior research, together with data from the present study, supports the notion that spiritual care helps patients to cope better with illness. Spiritual care is seen as providing a sense of direction, hope and inner peace, and allowing patients to accept and cope with problems, and to restore their sense of well-being and recovery through faith.

A study by Bailey, Moran and Graham (2009:43) supported the need to incorporate spirituality into nursing education. These scholars interviewed Irish nurses (*n* = 22) in order to understand their experience of providing spiritual care. A total of 77% agreed that spiritual care was part of their role, and 75% were of the opinion that spirituality had a role in nursing care and education. About 55% stated that making a personal connection was important for patients to be comfortable in expressing spiritual needs. However, spiritual care competence is required to provide for the needs expressed. Findings from the study by Bailey et al. (2009) resonate with the data in the present study. As seen in Figure 1, it included being there with the patient, giving hope, holding the patient’s hand, or spending time with the patient. A total of 82% of the sample in the study by Graham (2008:43) also described the importance of listening and 93% believed that spiritual care can be provided by allowing patients to discuss their fears, anxieties and concerns. Both studies emphasise the importance of listening closely to a patient in order to strengthen a spiritually sensitive relationship. Education should focus not only on this, but other spiritual interventions as part of holistic care.

There were several inquiries into whether such spiritual care is being integrated in nursing care and nursing education. A survey amongst nurses (*n* = 4054) in the United Kingdom by McSherry and Jamieson (2010:1757) found that they were aware that the provision of spiritual care enhanced the well-being of patients. They however felt that they needed more training and support to provide this level of care. These findings reinforce the need for nursing education to focus on listening to the patient, their family, friends and spiritual or religious leaders in order to identify the patient’s spiritual needs. A total of 82 participants in the sample indicated that spiritual care should be the collective responsibility of nurses, patients, family, friends and spiritual leaders, which reiterates that nurses should be prepared with education, knowledge and skill so as fulfil this responsibility. More than 90% of the participants in McSherry and Jamieson’ s (2010) also study reported this.

Ninety-one percent of the present sample and 95.5% of the sample from McSherry and Jamieson’ s (2010:1757) study said that they had encountered patients with spiritual needs. Participants of the present study stated that the spiritual needs of patients were identified by communicating with the patient (42.9%), relatives or friends (6.8%), spiritual or religious leaders (7.5%), other patients (2.1%), observing the patient (37.1%), and the nursing care plan (15.3%). Hence, nurses should be knowledgeable on how to gather information related to spiritual needs and on how to develop a nursing care plan.

### Practical implications

Spiritual care training should enhance the competencies of nurses, as well as the spiritual support that patients can be given during their illness (Vlasblom et al. 2010:790). According to Baldacchino (2006:892), nurses considered themselves incompetent because of a lack of preparation during their nursing education. Consequently, nurses recommended further continuing education to support their preparedness to deal with spirituality in nursing care.

Thus, nursing educators need to include a wide range of content and experiential learning in the basic nursing curriculum (Deal 2008:4). The spiritual aspects of patient are often overlooked as curricula have become crowded, with an emphasis on new technologies and care. Since 2000 there has however been a resurgence of interest in teaching spirituality and spiritual care to nursing students abroad, which suggests that South African educators should begin considering the same.

The strong support for the role of spirituality in nursing care and education may emanate from the fact that this was a mature sample with significant nursing experience. Their personal spirituality may have led to this, together with the fact that with experience comes a level of comfort in providing spiritually- based care. A significant proportion indicated that they had provided spiritual care despite having had no formal training for this. Spirituality requires specialised knowledge and skills to provide certain special spiritual care activities, as well as discerning between providing nursing care in a way that is ethical and meeting the primary need of patient physical care and well-being. Attention to the growing range of spiritual care activities that can be used as part of holistic care should therefore be considered in nursing education. Whilst it may not be possible to offer a specialised course on spirituality, a gradual interweaving of salient aspects should begin in current nursing education in South Africa.

## Limitations of the study

Data collection was confined to the province of KwaZulu-Natal, which may limit generalisability to the entire South African context. Despite poor participation at one hospital there was a high participation rate at the other hospitals. Furthermore, only questionnaires were used to collect data and perhaps interviews might have added richer data on what to teach specifically and the challenges associated with spiritual care.

## Recommendations

The study concluded that nurses needed greater academic preparedness related to spirituality and spiritual care. Attention should be focused on providing ethical interventions and on issues related to praying with the patient, spending time with the patient, supporting, reassuring and listening to the patient, and showing respect for the patient’s spiritual/religious beliefs. Referrals to spiritual or religious leaders should also be deliberated upon. These issues would obviate the barriers to providing spiritual care and the uncertainty on what and how to provide spiritual care. Further careful consideration by educators on what and how to teach is critical. An important starting point however is an earnest reflection on gradually interweaving it into current nursing education.

## Conclusion

Despite the potential for it to be interweaved into nursing education, especially given the void in the South African literature, spiritual care is undeniably an important part of a patient’s healing and restoration of hope and recovery. The patient profile is however rather different in South Africa, which warrants special consideration of diverse spiritualties and traditional healing interventions. Research that explores the role of spirituality in the context of patients’ lives is recommended so as to better understand what is needed when providing spiritual care. This study should be considered within the context of planning the new curriculum. For this to happen it is critical that there is a paradigm shift in nursing education and practice in South Africa; practitioners and educators should join the growing momentum abroad related to the provision of spiritually-based nursing care.

## Appendix A

### Appendix

**Questionnaire used during research**

Questionnaire on spirituality and spiritual care amongst professional nurses in KwaZulu-Natal

Dear participant

Thank you for your willingness to consider participating in this study. I am very grateful for same.

This survey is being conducted to explore spiritual care amongst professional nurses in KwaZulu-Natal. The survey first asks about the spiritual beliefs of nurses and then about the role of spirituality in nursing practice and nursing education. It includes the use of spiritually-based intervention. If you wish to comment on any question, please feel free to use the space in the margins. Your comments will be read and taken into account. You will need an average of 15 minutes to complete the questionnaire.

This survey uses a questionnaire that has been adapted from the instruments used by McSherry (2011) and Bhagwan (2002). Permission has been obtained from both researchers to utilise and adapt segments of their questionnaire for this study. Included in the questionnaire is a definition on spirituality to enable you to understand the term more clearly. Data will be collected from professional nurses who are working in provincial hospitals within KwaZulu-Natal. Ultimately it is hoped that the findings will enable the researcher to make recommendations to guide nursing education in relation to spirituality and spiritual care.

This project has been reviewed by the Faculty of Health Science Research and Higher Degrees Committee and has received ethical clearance from Durban University of Technology Institutional Research Ethics Committee.

Participation in the study is totally voluntary and you may withdraw from the study whenever you wish. Consent to participate is required and you will have to complete the attached consent form to indicate the same. All information received is confidential and your anonymity is guaranteed as your identifying details are not required. Kindly answer all questions. Should you have any queries kindly contact me at the number provided below or alternatively you can email me.

Kindly complete the consent form provided.

Thank you for your time and participation.

### PART A

The first section includes questions on demographic and various personal and professional background variables. Please indicate the appropriate response.

To aid you in responding to the following questions, respective definitions of spirituality and religion are provided.

- • Spirituality is defined as ‘the search for meaning, purpose, and connection with self, others, the universe, and ultimate reality, however one understands it. This may or may not be expressed through religious forms or institutions’.
- • Religion is defined as ‘an organized structured set of beliefs and practices shared by a community related to spirituality’.
- • When both spirituality and religion are referred to in one question, answer if either applies, or consider spirituality as inclusive of both religious and non-religious perspectives.

### PART B

The role of spirituality in nursing practice

The following questions ask your views about the role of spirituality in nursing practice.

Please rate your level of agreement or disagreement with each statement by circling the one number that best reflects your opinion on the five-point scale

**Table:**

	Strongly Disagree	Disagree	Uncertain	Agree	Strongly Agree
1. Spirituality is a basic aspect of being human.	1	2	3	4	5
2. Nurses should have more knowledge about spiritual care in nursing.	1	2	3	4	5
3. It is important for nurses to have knowledge about different religious faiths and traditions.	1	2	3	4	5
4. Religious concerns are outside of the scope of nursing practice.	1	2	3	4	5
5. Spiritual concerns are outside of the scope of nursing practice.	1	2	3	4	5
6. Nursing practice with a spiritual component has a better chance to empower clients than practice without such a component.	1	2	3	4	5
7. Knowledge of patients’ spiritual beliefs is important for effective nursing practice.	1	2	3	4	5
8. Nurses should be able to assess the positive or beneficial role of spiritual beliefs and practices in their patient’s lives.	1	2	3	4	5
9. Nurses should be able to assess the negative or harmful role of spiritual beliefs and practices in their patients’ lives.	1	2	3	4	5
10. The use of spiritual language and spiritual concepts in nursing practice are inappropriate.	1	2	3	4	5
11. The spiritual background of patients does not particularly influence nursing practice.	1	2	3	4	5
12. A nurse’s use of scripture or other spiritual texts in nursing practice are appropriate.	1	2	3	4	5
13. It is against nursing ethics to ever pray with a patient.	1	2	3	4	5
14. The use of spiritual concepts in nursing practice is inappropriate.	1	2	3	4	5
15. It is sometimes appropriate for a nurse to share his or her spiritual beliefs with a patient.	1	2	3	4	5
16. Addressing a patient’s spiritual beliefs is necessary for holistic nursing care.	1	2	3	4	5
17. Nursing education should include content related to spiritual diversity.	1	2	3	4	5
18. Nursing education should include content on how to deal with spiritual issues in nursing.	1	2	3	4	5

### Part C

**Spirituality and spiritual care**

For each question please circle one answer which best reflects the extent to which you agree or disagree with each statement.

**Table:**

	Strongly Disagree	Disagree	Uncertain	Agree	Strongly Agree
1. I believe nurses can provide spiritual care by arranging a visit by a hospital priest or the patient’s spiritual/religious leader.	1	2	3	4	5
2. I believe nurses can provide spiritual care by showing kindness, genuine concern and cheerfulness when giving care.	1	2	3	4	5
3. I believe spirituality is concerned with a need to forgive and a need to be forgiven.	1	2	3	4	5
4. I believe spirituality involves only going to church/place of worship.	1	2	3	4	5
5. I believe spirituality is not concerned with a belief and faith in a God.	1	2	3	4	5
6. I believe spirituality is about finding meaning in the good and bad events of life.	1	2	3	4	5
7. I believe nurses can provide spiritual care by enabling a patient to find meaning and purpose in their illness.	1	2	3	4	5
8. I believe spirituality is about having a sense of hope in life.	1	2	3	4	5
9. I believe spirituality has to do with the way one conducts one’s life here and now.	1	2	3	4	5
10. I believe nurses can provide spiritual care by spending time with a patient, giving support and reassurance in time of need.	1	2	3	4	5
11. I believe nurses can provide spiritual care by listening to and allowing patients time to discuss and explore their fears, anxieties and troubles.	1	2	3	4	5
12. I believe spirituality is a unifying force which enables one to be at peace with oneself and the world.	1	2	3	4	5
13. I believe spirituality does not include areas such as art, creativity and self-expression.	1	2	3	4	5
14. I believe nurses can provide spiritual care by having respect for privacy, dignity and religious and cultural beliefs of a patient.	1	2	3	4	5
15. I believe spirituality involves personal friendships and relationships.	1	2	3	4	5
16. I believe spirituality does not apply to those who are unsure of God or do not believe in God.	1	2	3	4	5
17. I believe spirituality includes peoples’ morals.	1	2	3	4	5

### PART D

**The role of spirituality in the lives of patients**

Listed below are several beliefs about patients’ spiritual capacity and the role of spirituality in their lives. Please rate your level of agreement or disagreement with each statement by circling the one number that best reflects your opinion on the five-point scale.

**Table:**

	Strongly Disagree	Disagree	Neutral	Agree	Strongly Agree
1. Hospitalisation is a time of spiritual awareness.	1	2	3	4	5
2 All patients have their own belief in spirituality.	1	2	3	4	5
3. Some patients do not have the mental abilities to reflect on spiritual matters.	1	2	3	4	5
4. Terminally ill patients search for meaning and purpose in life.	1	2	3	4	5
5. Spiritual participation helps protect the patient against depression.	1	2	3	4	5
6. It is not unusual for some patients to have spiritual experiences that influence their lives.	1	2	3	4	5
7 As patients grow older, they lose their natural connection to spirituality.	1	2	3	4	5
8. Some patients are exceptionally spiritually mature or gifted.	1	2	3	4	5
9. Some patients experience problems or anxiety due to spirituality that go unnoticed by nurses.	1	2	3	4	5
10. Religious beliefs provide guidelines for behaviours that are beneficial to patients.	1	2	3	4	5
11. As individuals grow, life experiences increase their spiritual maturity.	1	2	3	4	5
12. Patients who are abused or neglected may especially benefit from spiritual beliefs or practices.	1	2	3	4	5

### PART E

**Use of spiritual activities in patient care**

Please indicate the frequency of which you practice the following in your daily nursing care. Circle one most correct response for each question.

**Table:**

1. Gather information on the patient’s spiritual background.	Never	Rarely	Sometimes	Often
2. Assess a patient’s spiritual interest.	Never	Rarely	Sometimes	Often
3. Recommend spiritual books or writings.	Never	Rarely	Sometimes	Often
4. Pray privately for a patient.	Never	Rarely	Sometimes	Often
5. Pray or meditate with a patient.	Never	Rarely	Sometimes	Often
6. Recommend meditation.	Never	Rarely	Sometimes	Often
7. Use spiritual language or concepts.	Never	Rarely	Sometimes	Often
8. Help a patient verbalise their spiritual values.	Never	Rarely	Sometimes	Often
9. Recommend participation in a spiritual support system, programme, or activity.	Never	Rarely	Sometimes	Often
10. Recommend participation in volunteer social activities.	Never	Rarely	Sometimes	Often
11. Refer a patient to others for spiritual counselling, e.g., minister, priest, rabbi, chaplin or traditional healer.	Never	Rarely	Sometimes	Often
12. Recommend the use of a spiritual diary or journal.	Never	Rarely	Sometimes	Often
13. Recommend spiritual forgiveness, confession.	Never	Rarely	Sometimes	Often
14. Discuss with a patient the role of a spiritual belief system in relation to a significant others.	Never	Rarely	Sometimes	Often
15. Assist a patient to talk about their personal spiritual beliefs or practice.	Never	Rarely	Sometimes	Often
16. Help a patient consider the spiritual meaning of his/her current life situation.	Never	Rarely	Sometimes	Often
17. Help a patient reflect on his/her beliefsabout what happens after death?	Never	Rarely	Sometimes	Often
18. Help a patient reflect on his/her beliefs about death.	Never	Rarely	Sometimes	Often
19. Encourage a patient to discuss spiritual ritual as a practice intervention. (e.g., house blessings; remembering ancestors; celebrating life transitions; healing rituals).	Never	Rarely	Sometimes	Often
20. Participate in a patient’s spiritual rituals.	Never	Rarely	Sometimes	Often
21. Encourage patients to consider if spiritual beliefs and practices are helpful.	Never	Rarely	Sometimes	Often
22. Help patients to consider if spiritual beliefs and practices are harmful.	Never	Rarely	Sometimes	Often
23. Share your own spiritual beliefs or views.	Never	Rarely	Sometimes	Often
24. Collaborate with outside spiritual practitioners on behalf of patient.	Never	Rarely	Sometimes	Often
25. Encourage or recommend spiritual expression by the patient e.g. poetry, painting, or music.	Never	Rarely	Sometimes	Often
26. Listen to spiritual experiences or mystical experiences, reported by patient.	Never	Rarely	Sometimes	Often
27. Encourage the patient’s family to support any spiritual interest by the patient.	Never	Rarely	Sometimes	Often
28. Assess if the physical and social environment promotes or prevents the spiritual well-being of the patient.	Never	Rarely	Sometimes	Often

### PART F

**Spirituality and Nursing Education**
